# Maternal Knowledge and Awareness of Preventive Measures for Domestic Accidents Among Children in Jeddah, Saudi Arabia

**DOI:** 10.7759/cureus.71226

**Published:** 2024-10-10

**Authors:** Leenah M Alhadrami, Hamed S Habib, Balsam k Osman, Lama M Alqarni, Sarah F Almutiri, Shouq A Alhomoud

**Affiliations:** 1 Medicine, King Abdulaziz University Faculty of Medicine, Jeddah, SAU; 2 Pediatrics, King Abdulaziz University Hospital, Jeddah, SAU; 3 Medicine, King Abdulaziz University Hospital, Jeddah, SAU

**Keywords:** home accidents, home safety, maternal awareness, preventive measures, surveys

## Abstract

Background: Unintentional injuries are the leading cause of childhood injuries and result in thousands of deaths annually. However, children are at higher risk due to their increased curiosity and the time they spend at home. This study aimed to assess maternal knowledge regarding preventive measures for household injuries, including their frequency and severity.

Method: Using an electronic, self-administered questionnaire, this cross-sectional study assessed the knowledge and awareness of 433 mothers of children aged 1 to 12 years in our population between March 22 and July 31, 2023. Participants’ awareness levels were the main outcome.

Results: Of the participants, 54% reported that their children had experienced domestic injuries. The most frequently reported injuries were falls, burns, and wounds caused by sharp objects. Moreover, more than 50% of the children needed to visit the emergency department, of whom 10% required hospitalization. Regarding the awareness level score, 55% of mothers had moderate knowledge of home-related injuries. Furthermore, attending first-aid courses and other health education programs predicted higher awareness scores among the mothers.

Conclusions: More than half of the children in our study population had injuries that had occurred recently or in previous years. There was a significant discrepancy between the mothers' reported knowledge and their safety practices.

## Introduction

Injury is defined as physical damage to the human body due to exposure to excessive forces that exceed the physiological tolerance of the body [1]. Children are at higher risk of domestic accidents because they spend most of their time there and are less aware of dangers, more curious, and vulnerable to environmental threats [2,3]. Unintentional injuries are the most common cause of childhood injuries [4]. Thousands of children die due to accidents annually worldwide. Additionally, millions are admitted to hospitals for injuries resulting from accidents that could leave them permanently disabled [5]. Therefore, injuries remain the fifth-leading cause of death in infants and the number-one cause of death in children aged 1 to 19 years [6]. In Saudi Arabia, little is known about the frequency of home injuries and how they occur, which is a concern as emergency room visits have been increasingly reported [7]. Parents are often unaware of the scope of children's injuries and do not routinely consider the injury risk during their day-to-day interactions with their children. Understanding and practicing first aid is essential for child injury care because many negative consequences could be avoided [8,9]. A retrospective study conducted in Oman in 2020 reported that unintentional home accidents resulted in 1,333 children visiting the emergency department (ED) over six months, representing a prevalence of 7.7% among all children who went to the ED [10]. In 2011, a register-based cross-sectional analysis of 16 European countries showed that fatal home injuries were most common in children under five and rapidly declined thereafter [11]. Similarly, according to a 2021 cross-sectional study conducted in Al-Qassim, Saudi Arabia, among 250 mothers, 46.3% of their youngest children had previously experienced an injury at home [12]. Furthermore, a cross-sectional study involving 1,126 parents living in Ankara, Turkey, found that 13.8% of their children experienced at least one significant injury; moreover, their overall injury prevention score was low [13].

There is a significant number of unintentional home injuries among children. However, population-based studies evaluating this topic are lacking. Few studies have focused on maternal awareness of home accidents in our population.

This study aimed to assess maternal knowledge and awareness of preventive measures at home for unintentional injuries among children and to determine the frequency and severity of domestic injuries in children.

## Materials and methods

This cross-sectional study was conducted from March 22 to July 31, 2023, and included mothers of children aged 1 to 12 years. Mothers of children with debilitating diseases were excluded from the study. Among the 455 mothers who self-administered an electronic questionnaire on social media, 433 met the inclusion criteria.

The questionnaire used in this study was constructed and validated by the researchers. The first section included maternal characteristics and demographic data. The second section was designed for children injured in the present or previous years and included questions about domestic injuries and child characteristics. The third and fourth sections comprised 26 questions on home safety, maternal knowledge, and awareness and were designed to measure the participants' awareness levels.

The awareness level was categorized as follows: less than 50% (low awareness level), 50% to less than 75% (moderate awareness level), and 75% or higher (high awareness level).

This study was approved by the Unit of Biomedical Ethics Research Ethics Committee (REC), King Abdulaziz University. The procedures were in accordance with the ethical standards of the responsible committee on human experimentation (institutional or regional) and the Helsinki Declaration of 1975, as revised in 2000. Participants provided written informed consent before answering the questionnaire.

IBM SPSS Statistics for Windows, Version 21 (Released 2012; IBM Corp., Armonk, New York) was used for data analysis. Descriptive statistics are presented as frequencies and percentages (%). The chi-square test was used to assess the association between two qualitative variables. Stepwise multiple regression analysis was performed to analyze the factors affecting awareness levels among the studied mothers. Statistical significance was set at P < 0.05.

## Results

The demographics (Table 1) of the 433 mothers in this study revealed that 38.8% were aged 31-40 years, 20% had less than a college education, 70.9% had completed university, and 9% had postgraduate education. Of the participants, 50.6% were housewives and 91% were married. Among the mothers, 74.4% delivered their first child between the ages of 20 and 30 years, and 56.8% had ≥3 children. Furthermore, 235 (54.3%) mothers reported that their children had experienced domestic injuries in their present or previous years. Most of these children were <3 years (34.5%) and 3-6 years old (34%), and 58.3% were male. Among the children, 43.8% were the youngest, 22.1% were middle, and 34% were the oldest in their families. The most reported injuries were falls (58.7%), burns (17.4%), wounds caused by sharp objects (14.5%), and other injuries (5.1%). The most common cause of injury was child rioting (58.3%). Notably, 63.8% of the injured children visited the ED, and 10.2% required hospitalization. Approximately 21.3% of the injuries affected their daily lives, such as attending school or engaging in activities (Table 2).

**Table 1:** Characteristics of the participants in the study presented in numbers and percentages (N = 433)

Variables	Frequency	Percentage
Age		
Less than 20	3	0.7
20-30	110	25.4
31-40	168	38.8
More than 40	152	35.1
Number of children		
One	76	17.6
Two	111	25.6
Three or more	246	56.8
Number of people living in the house		
Less than 5	190	43.9
5 or more	243	56.1
Has your child been diagnosed with any disease or disorder?		
Yes	49	11.3
No	384	88.7
The last injury of one of your children was		
Not injured	198	45.7
Previous years	132	30.5
In this year	103	23.8

**Table 2:** The characteristics of the children who experienced an injury in this year or previous years, N = 235 (54.3%)

Variables	Frequency	Percentage
What kind of injury did your child have?		
Fall	138	58.7
Chemical poisoning	4	1.7
Burns	41	17.4
Electric shock	1	0.4
Wound with a sharp object	34	14.5
Animal bite	2	0.9
Swallow a coin	3	1.3
Other types of injury	12	5.1
Cause of injury?		
Child's rioting	137	58.3
Not following safety measures	32	13.6
Risky play	31	13.2
Parents' lack of attention	19	8.1
Babysitter is young	9	3.8
Not providing a safe home environment	7	3
Did the same injury happen in the past?		
No	191	81.3
Yes	44	18.7

Table 3 summarizes the age and sex distribution of the injured children according to injury type. Falls occurred more frequently in children aged ≤6 years (P = 0.01). There were no significant differences between sex and type of injury (P > 0.05); boys experienced falls and wounds caused by sharp objects at higher rates (34% and 10.2%, respectively) than did girls (24.7% and 4.3%, respectively). In comparison, girls experienced burns at a higher rate (9.4%) than did boys (8.1%).

**Table 3:** Age and gender distribution of the injured children according to the type of injury Statistical significance was determined using the chi-square test with a significance level set at p < 0.05.

Type of the injury	Gender of the child (N)		Age of child (N)			
	Female	Male	<3	3–6 years	>6-<9 years	9–12 years
Fall	58	80	48	55	18	17
Burns	22	19	19	10	3	9
Wound by a sharp object	10	24	7	11	6	10
Chemical poisoning	1	3	2	2	0	0
Swallow a coin	1	2	0	0	3	0
Animal bite	1	1	1	0	1	0
Leg fracture	1	1	0	0	0	2
Eye injury	1	1	0	0	0	2
Perfume drinking	0	1	1	0	0	0
Choking by food	1	1	2	0	0	0
Electrical shock	0	1	1	0	0	0
Bump on the head	0	1	0	1	0	0
Door being shut on their finger	1	0	0	0	1	0
Swallowed a medication	1	0	0	1	0	0
Nose bleeding	0	1	0	0	1	0
Total	98	137	81	80	34	40
Chi-square	0.195		0			
P	0.196		0.01			

**Figure 1:**
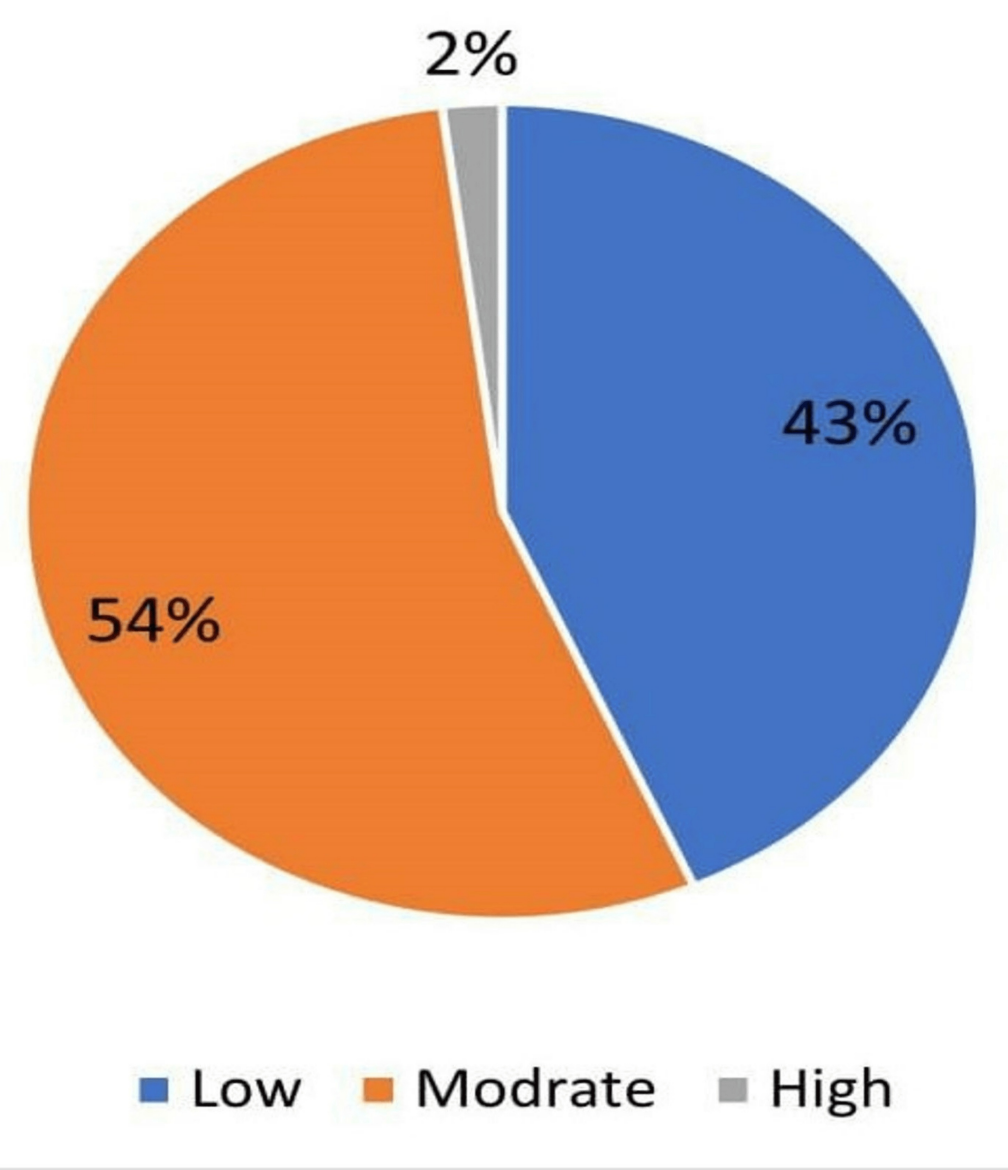
Knowledge and awareness levels of home safety among mothers

Overall, 55% of the mothers had moderate knowledge and awareness of home injuries, and 2% had a high level of awareness (Figure 1).

**Figure 2:**
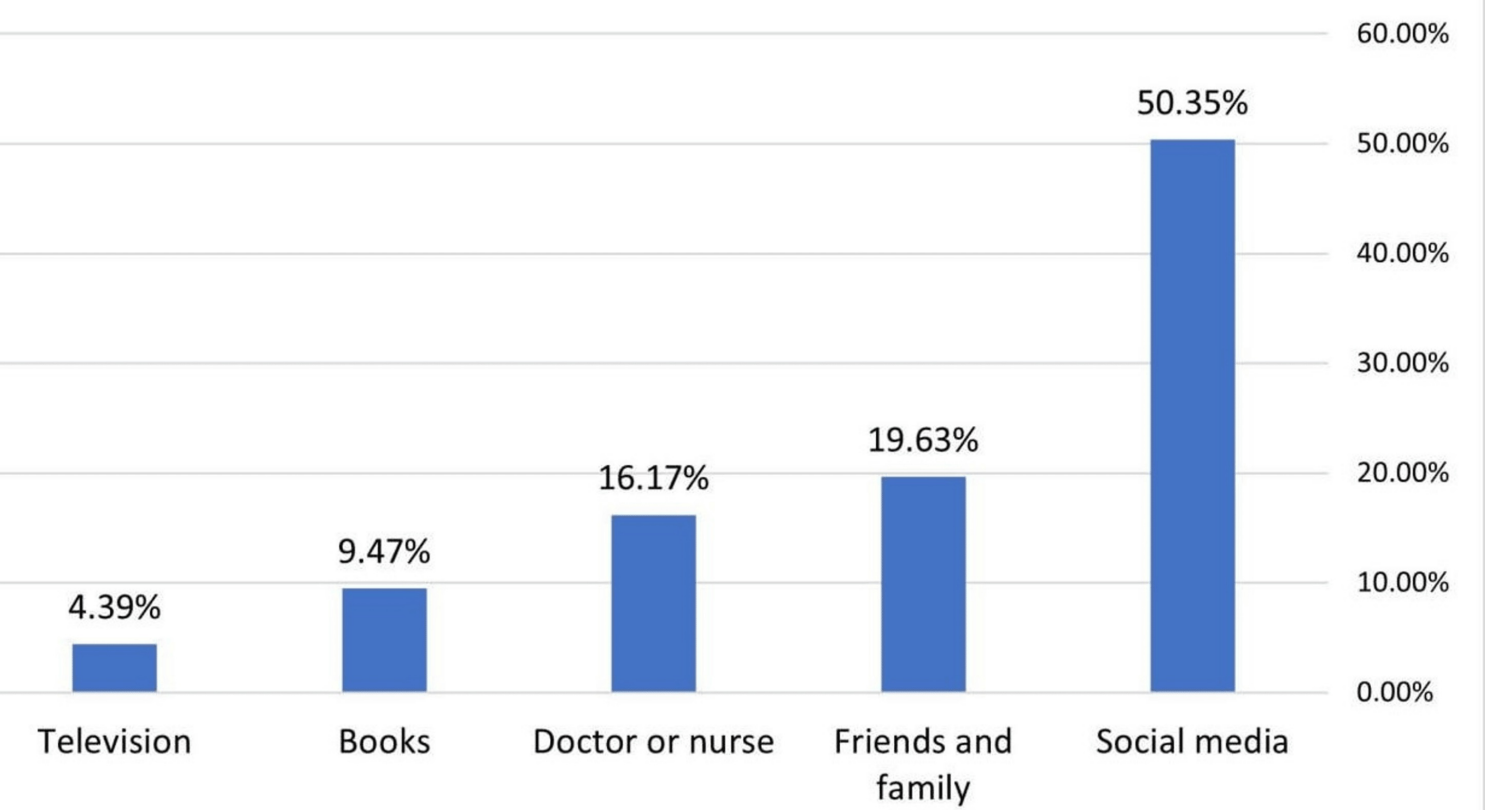
Sources of maternal health information The figure illustrates the sources of the health information reported by the participants. The percentages indicate the proportion of participants who obtained information on home safety from various sources.

Furthermore, 50.35% of the participants learned about home injuries from social media, 19.63% from friends and family, 16.17% from physicians or nurses, 9.47% from books, and 4.39% from television (Figure 2).

Multiple regression analysis revealed that attending first-aid courses and the source of health information were significant predictors of awareness (P < 0.001) (Table 4).

**Table 4:** Stepwise multiple regression analysis of factors affecting the awareness level of the studied mothers Dependent variable: awareness Level. "-" stands for excluded variables (no significant relation). Statistical significance was determined using the chi-square test with a significance level set at p < 0.05.

Variables	Beta	P	95.0% Confidence interval for beta
Have you ever attended a first-aid course	0.238	0.000	0.16 to 0.362
Sources of health information	-0.149	0.001	-0.108 to -0.026
The educational level	-0.017	0.725	-
Work	-0.034	0.488	-

Table 5 presents an overview of the types of injuries reported in 30 children with various diseases and highlights that the occurrence of injuries did not differ significantly among the different diseases in this study.

**Table 5:** Type of injury in relation with the child disease ADHD: attention deficit hyperactivity disorder, G6PD: glucose-6-phosphate dehydrogenase. Statistical significance was determined using the chi-square test with a significance level set at p < 0.05.

Diseases	What kind of injury did your child have?							Total
	Fall	Burns	Wound by a sharp object	Eye injury	Perfume drinking	Door being shut on their finger	Nose bleeding	
ADHD	5	0	2	0	0	0	0	7
Asthma and lactose intolerance	0	0	0	0	1	0	0	1
Asthma and peanut allergy	0	1	0	0	0	0	0	1
Astigmatism	0	0	0	0	0	1	0	1
Asperger syndrome	1	0	0	0	0	0	0	1
Born with one ear	0	0	1	0	0	0	0	1
Febrile seizure	1	0	0	0	0	0	0	1
celiac disease	0	1	0	0	0	0	0	1
Asthma	1	0	0	1	0	0	1	3
G6PD deficiency and anemia	1	0	0	0	0	0	0	1
Low oxygen at birth	1	0	0	0	0	0	0	1
Alopecia	1	0	0	0	0	0	0	1
Epilepsy	1	0	0	0	0	0	0	1
Sickle cell anemia	1	1	0	0	0	0	0	2
Diabetes mellitus type 1	1	0	0	0	0	0	0	1
Autism	0	0	2	0	0	0	0	2
Developmental delay	0	1	0	0	0	0	0	1
G6PD	0	1	0	0	0	0	0	1
Sensorineural hearing loss	1	0	0	0	0	0	0	1
Intracerebral hemorrhage	1	0	0	0	0	0	0	1
Total	16	5	5	1	1	1	1	30
Chi-square	0.255							
P value	1.00							

There was a significant association between awareness and the mothers’ age at first childbirth (P = 0.045). Other maternal characteristics, such as age (P = 0.938), educational level (P = 0.54), employment status (P = 0.53), marital status (P = 0.767), monthly income (P = 0.313), number of children (P = 0.541), and number of people living in the house (P = 0.130), were not significantly related to the awareness level.

## Discussion

In this study, we aimed to determine the mothers' understanding and awareness of safety measures to prevent unintentional injuries in children. Additionally, we assessed the frequency and severity of domestic injuries in children.

Our study demonstrated that most mothers were aged 31-40 years and were highly knowledgeable, with a significant proportion having completed higher education. Notably, a significant proportion were housewives, indicating the impact of maternal presence on the home environment. This finding is consistent with a study conducted in Egypt of 270 rural mothers regarding children with home-related injuries. The study revealed that most mothers were housewives and reported more child injuries (69.3%) [14]. Similar results have been reported by Al-Bshri et al. [12]. However, this could also be attributed to the lower prevalence of working mothers in these areas.

Furthermore, in our study, no statistically significant relationship existed between awareness level and the mothers' educational background or occupational status. However, a study conducted in Makkah reported that working mothers and those with a higher education level demonstrated better knowledge and attitudes regarding proper practices [15]. Conversely, another study revealed an inverse relationship between working mothers with higher levels of education and knowledge of injury prevention [16]. The level of maternal awareness may vary depending on cultural and societal factors such as cultural norms, societal expectations, and access to resources and information related to home safety.

In the present study, we found that more than 50% of the mothers reported that their children had experienced an injury at home, either in the present or in previous years. Of note, most mothers included in this study had three or more children. Moreover, our study findings are consistent with those of a study conducted in Egypt [17]. That study found that family size, number of children, and living with a single mother did not directly influence domestic accident awareness levels. This could be explained by the fact that mothers may have gained experience and become more familiar with the risks and safety measures associated with domestic accidents.

Regarding injury type, we found that the most common injuries were falls, burns, and cuts from sharp objects, most occurring in children under six years of age, suggesting that young age groups are vulnerable. Additionally, the majority were boys, which may indicate sex-related risk-taking behavior. Consistent with the study by Lasi et al. [18], our study found that girls had more instances of burns. However, falls were more likely among Mozambican boys (65%) [19].

This finding agrees with a study showing that the most common injuries in children were falls and burns [20]. Other studies have reported similar findings [12,14,21], highlighting the need for preventive measures such as childproofing homes and increasing awareness and supervision. This would help reduce the prevalence of preventable injuries caused by these injury patterns.

A significant proportion of injured children required medical attention, with the majority requiring emergency department (ED) visits and a minor percentage needing hospitalization. This indicates the severity and importance of awareness of domestic injuries in children. Additionally, it could pose a burden on the healthcare system.

The current study showed that 97% of the participants had low-to-moderate awareness regarding preventive measures at home for unintentional injuries. Similar results were found in a study in Riyadh, where 88% had poor-to-good practices related to knowledge and awareness of unintentional home injury [22]. This difference may be due to the different sample sizes and study populations.

Our results revealed that burns were more common in children younger than three years. This is consistent with the study conducted by Paes et al., which demonstrated that burn injuries primarily affected children between the ages of 1 and 4 years, as they had the lowest accident risk awareness [3].

The present study revealed that 50% of the participants chose social media as the source of their knowledge about home-related injuries. This is consistent with a study conducted in Egypt, which showed that 43.3% of the participants gained their knowledge from social media [14]. Only 4% of our study participants gained knowledge from television. However, 38.5% of women who participated in a study by Kamel et al. learned about first aid from television [23]. Various demographic characteristics of the population may explain this.

Our study showed no statistically significant relationship between maternal awareness levels and age (P = 0.938). However, a study conducted in Egypt found that younger mothers had higher knowledge, attitudes, and practice scores [14]. Moreover, mothers who had their first child at an older age had higher awareness levels than those who had their first child at a younger age. Another study conducted in Baghdad City showed that mothers' knowledge improved with age or as they had more children, which can be explained by the fact that mothers gain various experiences over time [16].

Although other studies [24-17] have found a significant positive correlation between socioeconomic status and either attitudes or knowledge of mothers toward home injury, this study did not find such a relationship.

Furthermore, having a child with a diagnosed disease or disorder did not significantly affect maternal awareness levels, suggesting that the information provided to the mothers was similar and comprehensive regardless of their children's specific conditions. This finding is consistent with that of other studies [15-25], indicating higher awareness levels among mothers who attended a first-aid course compared with those who did not. This highlights the importance of educational programs and training to improve maternal awareness of child safety.

Our study has several limitations. First, there may have been recall bias, as mothers may have had difficulties accurately recalling past events or experiences.

Second, as a cross-sectional study, our research design did not allow for the establishment of causality between variables. Although these associations were identified, we could not determine the direction of causality or rule out potential confounders.

Finally, our study focused on mothers' knowledge of and attitudes toward home safety. However, considering the broader context of caregiving and household safety, it is crucial to recognize the roles of both parents.

## Conclusions

In conclusion, more than half of the children in our study population had injuries that occurred recently or in previous years. There was a remarkable difference between the mothers' reported knowledge and their safety practices. Accordingly, implementing well-designed safety intervention methods, such as programs that guide the mothers of young children regarding the most important domestic accidents, how to prevent them, and the necessary measures to address them, is crucial.

As most mothers gain knowledge from social media, healthcare providers could use it as a platform to spread awareness of accidents, their potential impact, and strategies for creating a safe environment to prevent such incidents. Additionally, primary healthcare facilities could play an effective role in prevention by providing first-aid instructions and regularly disseminating awareness among caregivers.
